# Impact of bariatric surgeries on bone density in patients with severe obesity

**DOI:** 10.1186/s12893-026-03572-1

**Published:** 2026-03-18

**Authors:** Ahmed Mohammed Salah Eldeen Othman Elansary, Mohamed Hassan Ali Fahmy, Mostafa Zaghloul, Ahmed Mohammed Abdelsalam, Aya H. Eldesouky, Ehab Fathy

**Affiliations:** 1 General Surgery Department, Faculty of Medicine, Cairo University, Cairo, Egypt; 2 Rheumatology and Rehabilitation Department, Faculty of Medicine, Cairo University, Cairo, Egypt

**Keywords:** Bariatric surgeries, Severe obesity, Bone mineral density, Dual-Energy X-ray Absorptiometry (DXA)

## Abstract

**Introduction:**

Bariatric surgery is approved to promote weight loss and induce remission of obesity-related medical conditions. However, the impact of these procedures on bone density is still debated. This study aimed to assess the effect of bariatric surgeries on bone mineral density using dual-energy X-ray absorptiometry (DXA) scan in sleeve gastrectomy and bypass surgery.

**Methods:**

The prospective cohort study recruited 32 patients with severe obesity, who underwent metabolic and bariatric surgery. Patients were divided into two groups and monitored for one year. Laparoscopic sleeve gastrectomy group included 18 patients; the Bypass group included 14 patients. The primary outcome was the assessment of bone density by DXA scan. Secondary outcomes included serum calcium, parathyroid hormone, and vitamin D levels.

**Results:**

Patients who underwent gastric bypass surgery had a higher incidence of bone loss at the femoral neck (*p* = 0.030) and radius (*p* = 0.043) compared to those who underwent sleeve gastrectomy. In the sleeve gastrectomy group, bone density at the spine was significantly reduced at one year postoperatively, while no statistically significant change was observed in the gastric bypass group. Vitamin D level was significantly higher in the sleeve gastrectomy group than in the bypass group (*p* = 0.029). Patients compliant with medications demonstrated significantly less bone loss with higher calcium and vitamin D and lower parathyroid hormone levels compared to noncompliant patients (*p* < 0.05).

**Conclusion:**

Metabolic and bariatric surgery is associated with changes in bone mineral density, most notably after gastric bypass procedures, accompanied by reductions in calcium and vitamin D levels and increased parathyroid hormone. These findings underscore the importance of early postoperative monitoring of bone health using DXA and biochemical markers, particularly after bypass procedures and in patients with poor adherence to supplementation.

## Key points

Metabolic and bariatric surgery impacts bone mineral density, especially after gastric bypass.

Sleeve gastrectomy is associated with less bone loss compared to gastric bypass.

Patient compliance with calcium and Vitamin D supplementation reduces bone loss risk.

## Introduction

 Numerous chronic illnesses associated with severe obesity negatively impact overall health and increase the likelihood of death among individuals with obesity. A high body mass index (BMI) significantly increases the risk of obesity-related diseases, such as obstructive sleep apnea, type 2 diabetes, and hypertension (HTN) [1, 2].

Various metabolic and bariatric surgical techniques differ significantly in their effectiveness with regard to perioperative outcomes, associated medical problems reduction, and weight loss. Roux-en-Y gastric bypass (RYGB) and sleeve gastrectomy (SG) are two common bariatric procedures [3]. Both SG and RYGB have benefits and drawbacks of their own. Sleeve gastrectomy has quickly become a popular Metabolic and bariatric surgery due to its technological ease [4, 5].

However, there are growing concerns about the negative effects of Metabolic and bariatric surgery on bone health, including bone loss, increased fragility, and an increased risk of fracture following the procedure [6]. Dual-energy X-ray absorptiometry (DXA) measurements can provide valuable information for evaluating postoperative bone outcomes and addressing potential issues [7].

Bone mineral density (BMD), as assessed by dual-energy X-ray absorptiometry, is a key predictor of fracture risk, whereas clinical fractures are diagnosed using conventional radiographic imaging. However, research indicates that BMD is used to diagnose approximately half of clinical fractures in women without osteoporosis (i.e., those with a BMD T-score greater than − 2.5) [8]. The BMD is often higher than normal in people with type 2 diabetes [9].

Furthermore, most individuals with fragility fractures have bone mineral densities (BMDs) that are within the normal range or show only slight bone mass loss. This indicates that maintaining bone structure quality is more important than maintaining bone mass [10]. In addition, research findings on the impact of SG and RYGB on bone loss are conflicting [11–13]. Therefore, this study aimed to assess the effect of Metabolic and bariatric surgery on bone mineral density using DXA scans in patients undergoing sleeve gastrectomy or bypass surgery.

Despite growing evidence linking metabolic and bariatric surgery to alterations in bone metabolism, current literature remains inconsistent regarding the comparative impact of sleeve gastrectomy and Roux-en-Y gastric bypass on bone mineral density. Many available studies are limited by short follow-up durations, heterogeneous patient populations, or reliance on surrogate biochemical markers rather than direct measures of skeletal integrity. The present study adds novel insight by directly comparing postoperative changes in BMD between SG and RYGB using standardized DXA assessments in a single-center cohort with consistent surgical and follow-up protocols. By controlling for confounding variables such as age, sex, comorbidities, and baseline bone density, this investigation provides a clearer understanding of how different metabolic and bariatric surgery uniquely influence bone health. This focused approach aims to help clinicians make more informed decisions when balancing the metabolic benefits of surgery with potential skeletal risks.

A major strength of this study lies in its use of dual-energy X-ray absorptiometry (DXA), the gold-standard imaging modality for evaluating bone mineral density. DXA offers high precision, low radiation exposure, and reproducible quantitative data, allowing for reliable detection of subtle bone loss that may occur after metabolic and bariatric surgery. Unlike serum biomarkers or clinical risk assessments, DXA provides site-specific measurements—particularly at the lumbar spine and femoral neck. Its ability to track longitudinal changes enhances the accuracy of evaluating postoperative skeletal outcomes and minimizes measurement variability. Given the inconsistent findings in the literature regarding the effects of different bariatric procedures on bone health, this study aimed to compare changes in bone mineral density following sleeve gastrectomy and gastric bypass using standardized DXA assessments.

## Materials and methods

### Study design, setting, and location

This prospective cohort study was conducted at the General Surgery Department, University Hospitals. Patient recruitment and surgical procedures were performed between August 2023 and August 2024. Postoperative follow-up was completed at 12 months for all patients, with final data collection concluding in August 2025.

### Eligibility criteria

The study included data from patients of both genders, adult patients aged 18 years or older, with a body mass index (BMI) > 35 kg/m² without associated medical problems or ≥ 30 kg/m² with associated medical problems. These patients were managed using laparoscopic sleeve gastrectomy or gastric bypass surgery under general anesthesia. Patients who had suffered from bone metabolism abnormalities, or severe degenerative changes or fracture deformity in the measurement area or exhibited severe mental or cognitive disorders were excluded from the study. Moreover, children aged less than 10 years, pregnant patients and patients who were not suitable for general anesthesia were excluded from the study.

**Fig. 1:**
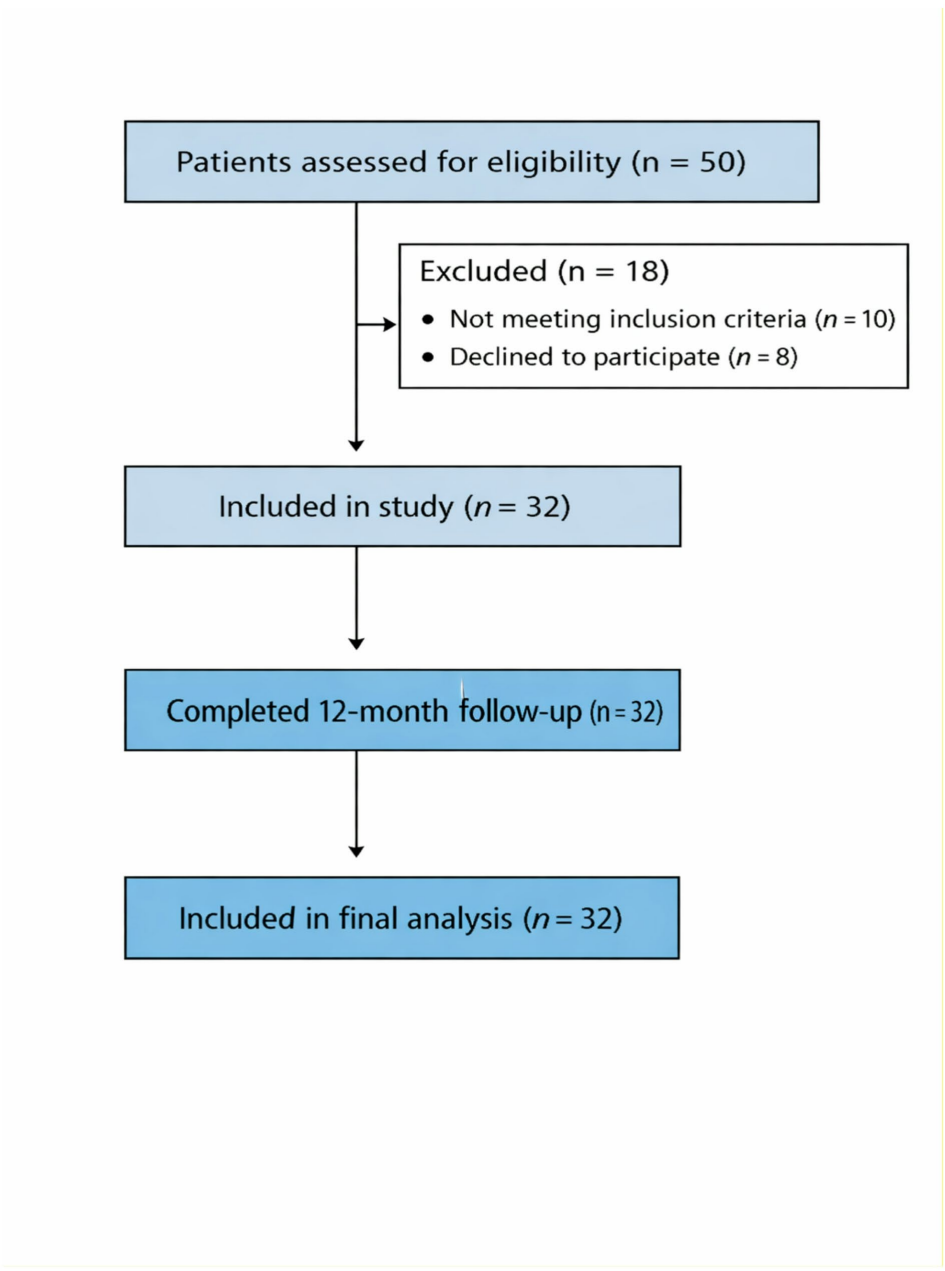
STROBE flow diagram of participant enrollment and follow-up

Patients were enrolled consecutively, and all included participants completed a planned 12-month postoperative follow-up, defined as assessments performed within ± 4 weeks of the 12-month time point. (Fig. 1).

### Ethical considerations

The study protocol was reviewed and approved by the Institutional Ethics Committee of the Faculty of Medicine, Cairo University (Approval No.: MD-421–2023). All procedures were conducted in accordance with the Declaration of Helsinki and its later amendments. Written informed consent was obtained from all participants prior to enrollment, and the confidentiality of participant data was strictly maintained.

### Preoperative assessment

A thorough evaluation was performed to assess the patients’ general health, associated medical problems, risk factors, psychological condition, and capacity to follow a postoperative regimen. Laboratory and radiographic tests were performed, including an upper gastrointestinal endoscopy, a chest X-ray, and an abdominal ultrasound. Cardiovascular and endocrine assessments were also performed, including echocardiography, electrocardiography, and pulmonary function testing.

### Preoperative DXA scan

Before the surgery, a DXA scan was done to assess the bone density. Patients were advised not to take calcium supplements on the day before the examination. Premenopausal patients were asked about the possibility of pregnancy, and a pregnancy test was performed prior to the examination when indicated. Patients should avoid metal objects and wear loose-fitting clothing.

The patient was positioned on a specialized X-ray table with the assistance of a technician. The technician passed a scanning arm over the patient’s body, capturing photographs of his bones. Technicians scan patients’ hips, spine, and extremities. A bone density scanner uses two forms of low-level radiation to convert bone density into computer images and graphs. Bone appears radiopaque relative to soft tissue. In the background of the test images, fat, muscles, and other soft tissue appeared as black shadows.

### Interpretation

Bone mineral density is the standard for diagnosing osteoporosis. Changes in BMD during the follow-up are calculated using g/cm^2^.

In patients older than 50 years without fractures, osteoporosis is diagnosed if the BMD T-score falls below a diagnostic threshold at any site. According to the World Health Organization (WHO), a T-score greater than or equal to −1.0 is considered normal. A score between − 1.0 and − 2.5 indicates osteopenia. A score of less than or equal to −2.5 indicates osteoporosis [14].

In premenopausal women, the International Society for Clinical Densitometry has recommended Z-scores as a metric for the classification of BMD readings. This approach enables the comparison of women with a reference population that is matched in terms of age. Young women are classified as having BMD that is “below the expected range for age” if their Z-score is less than 2.0. Conversely, those with Z-scores greater than 2.0 are categorized as having BMD “within the expected range for age.” In the absence of factors such as aging or menopause, a low Z-score suggests the presence of an underlying secondary condition [15, 16].

Bone mineral density was assessed using dual-energy X-ray absorptiometry (DXA) (Hologic Discovery system, Hologic Inc., Bedford, MA, USA) with manufacturer-provided software. Daily quality control was performed using a standard phantom according to the manufacturer’s recommendations.

Site-specific precision error, expressed as coefficient of variation (CV%), was based on institutional quality control data and was approximately 1.0% for the lumbar spine, 1.5% for the femoral neck, and 1.8% for the forearm radius. Least significant change (LSC) was calculated as 2.77 × precision error, corresponding to thresholds of approximately 2.8% for the lumbar spine, 4.2% for the femoral neck, and 5.0% for the forearm radius.

### Operative details

Patients were divided into two groups. The laparoscopic sleeve gastrectomy (LSG) group included 18 patients, and the bypass group included 14 patients who underwent gastric bypass (4 patients underwent revisional gastric bypass surgery after LSG, 2 patients underwent revisional gastric bypass surgery after vertical banded gastroplasty surgery, 4 patients underwent primary laparoscopic one-anastomosis gastric bypass (OAGB), and the remaining 4 patients underwent primary LRYGB surgery).

A comprehensive evaluation encompassing dietary practices, reflux symptoms, prior Metabolic and bariatric surgery, patient desire, and compliance was conducted to ascertain the most suitable Metabolic and bariatric surgery for each patient.

The administration of general anesthesia was a common practice in Metabolic and bariatric surgery, including laparoscopic revisional bypass surgery, laparoscopic RYGB, laparoscopic sleeve gastrectomy, and laparoscopic one anastomosis gastric bypass (OAGB). Following the implementation of Enhanced Recovery After Surgery (ERAS) protocols, the patient was transitioned to a standard ward, subject to rigorous surveillance.

Laparoscopic sleeve gastrectomy was performed using a standardized technique over a bougie size of 36–40 Fr, starting approximately 4–6 cm proximal to the pylorus. Staple-line reinforcement was performed according to surgeon preference. Hiatal hernia repair was undertaken when a clinically significant hernia was identified intraoperatively.

For gastric bypass procedures, Roux-en-Y gastric bypass or one anastomosis gastric bypass was performed with standardized limb lengths. For RYGB, the biliopancreatic limb was 100 cm and the alimentary limb 100 cm. For OAGB, the biliopancreatic limb was 150–200 cm. Limb lengths were selected based on patient characteristics and institutional protocol.

Revisional gastric bypass procedures were performed for indications including inadequate weight loss, weight regain, or procedure-related complications following prior bariatric surgery. The interval between the primary procedure and revision was at least 12 months in all revisional cases.

The gastric bypass group comprised both primary and revisional procedures, including laparoscopic Roux-en-Y gastric bypass (LRYGB) and one anastomosis gastric bypass (OAGB). Revisional cases included patients converted from prior sleeve gastrectomy or vertical banded gastroplasty. Due to the limited sample size, subgroup analyses based on primary versus revisional surgery or bypass technique were not powered to detect statistically significant differences; therefore, all bypass procedures were analyzed collectively. This approach was chosen to explore overall trends in bone mineral density changes associated with bypass-type procedures compared with sleeve gastrectomy.

### Postoperative care

Patients were discharged on the first postoperative day, having received the pertinent postoperative instructions. No patients needed re-examination or a prolonged hospital stay. The patients were subsequently followed up at one week, one month, three months, six months, and one year later. All patients were advised to take daily oral supplements, including calcium citrate 665 mg twice per day for 1 year and 300,000 IU of vitamin D supplements once per month for at least 6 months. One year postoperatively, all patients were evaluated with follow-up labs including Vitamin D, Serum Ca levels, serum parathyroid hormone (PTH) levels, and DXA scan.

Medication adherence was assessed during follow-up visits using patient self-report obtained through structured clinical interviews. Patients were classified as compliant if they reported regular adherence to the prescribed calcium and vitamin D supplementation regimen throughout the follow-up period. Assessed by structured patient interview; no objective verification (e.g., pill counts) was performed.

### Study outcomes

The primary outcome was the quantitative change in bone mineral density, assessed by DXA as absolute BMD (g/cm²) and corresponding T-score or Z-score values, measured preoperatively and at one year postoperatively. Categorical classification of bone loss was used as a secondary descriptive analysis.

The prespecified primary endpoint was the change in bone mineral density at one year as assessed by DXA. Secondary outcomes included biochemical markers and categorical bone loss classification.

Secondary outcomes included serum calcium and vitamin D levels.

Percent excess weight loss (%EWL) was not prespecified as a study outcome and therefore was not included in the analysis.

Weight loss was not prescribed as an intervention in this study but occurred as a consequence of bariatric surgery. Weight-related outcomes were therefore reported descriptively using percent total weight loss (%TWL), which was prespecified as the weight metric. Percent excess weight loss was not calculated.

### Sample size

As indicated by Misra et al. [17], the BMD of the hip, prior to metabolic and bariatric surgery, was 1.22 ± 0.03 as determined by DXA. Subsequent to the surgical intervention, a decline to −0.10 was observed. The sample size, calculated to assess the impact of metabolic and bariatric surgery on bone mineral density across diverse age groups and body mass indices, employing DXA scans, was determined to be 28 metabolic and bariatric surgery patients, with a follow-up rate of 1%. The Clinical Sample Size Calculator is employed, with the parameters set at 0.05 alpha error, 0.80 power, and a confidence interval of 95%.

The sample size calculation was based on detecting a clinically meaningful within-group change in bone mineral density from baseline to one year following bariatric surgery, as reported in prior literature. The study was not specifically powered to detect between-group differences between sleeve gastrectomy and gastric bypass procedures. Accordingly, comparisons between surgical groups were considered exploratory.

### Statistical analysis

The statistical analysis of the data was conducted using IBM SPSS Statistics, version 21.0 (IBM Corp., Armonk, NY, USA). The numerical values and percentages were used to display the categorical data. The Shapiro-Wilk test was employed to evaluate the normality of continuous data. Quantitative data were reported using range, mean, and standard deviation (SD). The chi-square test and Fisher’s exact test were employed to assess the association between two sets of qualitative variables. Within-group comparisons of preoperative and postoperative measurements were performed using paired statistical tests (paired t-test for normally distributed data or Wilcoxon signed-rank test for non-normally distributed data). Between-group comparisons between the sleeve gastrectomy and gastric bypass groups were conducted using independent-samples tests (independent t-test or Mann–Whitney U test, as appropriate).

An exploratory 2 × 2 mixed-design ANOVA was additionally performed for each skeletal site, with surgical group as the between-subjects factor and time (preoperative vs. postoperative) as the within-subjects factor. The Group × Time interaction term was examined to assess differential change between groups. Given the limited sample size, these analyses were considered exploratory and interpreted cautiously.

## Results

A total of 40 patients were assessed, and eight of them were excluded. One patient was pregnant, and seven did not meet the inclusion criteria. The remaining 32 patients were divided into two groups: 18 underwent laparoscopic sleeve gastrectomy and 14 underwent gastric bypass. There were no significant differences in the demographic characteristics of the groups concerning age, gender, medical history, or previous metabolic and bariatric surgery (Table 1).

**Table 1:** Patients’ baseline characteristics (*n* = 32)

		LSG (*n* = 18)	Gastric bypass (*n* = 14)	*P* value
Age, years	**Mean ± SD**	35.67 ± 9.85	40.14 ± 8.61	0.73
	***P *** **value**	0.882	0.72	
Gender, n (%)	**Male**	3 (16.7%)	1 (7.1%)	0.419
	**Female**	15 (83.3%)	13 (92.9%)	0.413
Medical history, n (%)	**HTN**	8 (44.4%)	2 (14.3%)	
	**DM**	0	2 (14.3%)	
	**None**	10 (55.6%)	10 (71.4%)	
Previous bariatric surgery, n (%)	**sleeve**	0	4 (28.6%)	
	**VBG**	0	2 (14.3%)	
	**None**	18 (100%)	8 (57.1%)	

*LSG* Laparoscopic Sleeve Gastrectomy, *n* number, *SD* Standard deviation, *DM* Diabetes mellitus, *HTN* Hypertension, *VBG* Vertical banded gastroplasty

Although baseline differences between groups were not statistically significant, the gastric bypass group showed a slightly higher mean age and BMI and included two patients with type 2 diabetes mellitus, whereas no patients in the sleeve gastrectomy group had diabetes.

A sensitivity analysis excluding patients with type 2 diabetes mellitus did not materially alter the observed pattern of bone mineral density changes between the two surgical groups; however, these analyses were underpowered due to the small sample size.

Exploratory comparison within the gastric bypass group did not reveal statistically significant differences in postoperative bone loss between primary and revisional procedures; however, these analyses were underpowered and should be interpreted cautiously.

In the spine scan, a significant reduction in bone mineral density was observed in the sleeve gastrectomy group when comparing preoperative and postoperative measurements (*p* = 0.03). In contrast, the gastric bypass group did not demonstrate a statistically significant pre–post change (*p* = 0.14). The intergroup comparison showed no significant difference in postoperative spine bone loss between the two groups (*p* = 0.56). In the femoral neck and radius scan, postoperative bone loss was significantly higher in gastric bypass patients compared to their preoperative DXA scans (*p* = 0.013 and 0.04, respectively) and compared to the sleeve gastrectomy group (*p* = 0.030 and 0.043, respectively; Table 2).

**Table 2:** Pre and postoperative Dual-energy X-ray absorptiometry (DXA) scan (*n* = 32) Within-group comparisons reflect preoperative versus postoperative changes, while between-group comparisons assess differences between sleeve gastrectomy and gastric bypass

*n* (%)		LSG (*n* = 18)	Gastric bypass (*n* = 14)	*P* value
Preoperative spine	Bone loss	0 (0.0%)	0 (0.0%)	1.00
	Normal	18 (100%)	14 (100%)	
Postoperative spine	Bone loss	4 (22.2%)	2 (14.3%)	0.56
	Normal	14 (77.8%)	12 (85.7%)	
	**Within-group ** ***P*** **value**	0.03*	0.14	
Preoperative femoral neck	Bone loss	0 (0.0%)	0 (0.0%)	1.00
	Normal	18 (100%)	14 (100%)	
Postoperative femoral neck	Bone loss	1 (5.6%)	5 (35.7%)	0.030*
	Normal	17 (94.4%)	9 (64.3%)	
	**Within-group ** ***P*** **value**	0.31	0.013*	
Preoperative forearm (radius)	Bone loss	2 (11.1%)	2 (14.3%)	0.78
	Normal	16 (88.9%)	12 (85.7%)	
Postoperative forearm (radius)	Bone loss	3 (16.7%)	7 (50%)	0.043*
	Normal	15 (83.8%)	7 (50%)	
	**Within-group ** ***P*** **value**	0.63	0.04*	

*n* number

^*^Significant at *p* < 0.05

Quantitative analysis of DXA measurements demonstrated a significant mean reduction in spine BMD in the sleeve gastrectomy group at one year compared to baseline, whereas the gastric bypass group showed a non-significant change. At the femoral neck and forearm radius, the gastric bypass group exhibited a greater mean decline in BMD compared to the sleeve gastrectomy group. These findings were consistent with the categorical bone loss analysis. (Table 3).

**Table 3:** Preoperative and postoperative DXA-derived bone mineral density (BMD) and T-scores after laparoscopic sleeve gastrectomy and gastric bypass (*n* = 32). Within-group *P* values represent preoperative versus postoperative comparisons. Between-group *P* values represent comparisons between laparoscopic sleeve gastrectomy and gastric bypass groups

**Lumbar spine**			
Mean ± SD	LSG (*n* = 18)	Gastric bypass (*n* = 14)	*P* value
Preoperative BMD (g/cm²)	1.18 ± 0.09	1.16 ± 0.08	0.520
Postoperative BMD (g/cm²)	1.10 ± 0.11	1.12 ± 0.10	0.480
Within-group P value	0.030*	0.140	
Preoperative T-score	0.4 ± 0.6	0.3 ± 0.5	0.610
Postoperative T-score	−0.7 ± 0.8	−0.5 ± 0.7	0.570

Femoral neck			
Variable	LSG (n = 18) Mean ± SD	Gastric bypass (n = 14) Mean ± SD	*P* value
Preoperative BMD (g/cm²)	1.02 ± 0.07	1.01 ± 0.06	0.670
Postoperative BMD (g/cm²)	0.99 ± 0.08	0.91 ± 0.09	0.028*
Within-group P value	0.310	0.013*	
Preoperative T-score	0.2 ± 0.5	0.1 ± 0.6	0.710
Postoperative T-score	−0.3 ± 0.7	−1.3 ± 0.8	0.030*

Forearm (radius)			
Variable	LSG (n = 18) Mean ± SD	Gastric bypass (n = 14) Mean ± SD	*P* value
Preoperative BMD (g/cm²)	0.78 ± 0.06	0.77 ± 0.07	0.740
Postoperative BMD (g/cm²)	0.76 ± 0.07	0.69 ± 0.08	0.041*
Within-group *P* value	0.630	0.040*	
Preoperative T-score	−0.5 ± 0.7	−0.6 ± 0.8	0.810
Postoperative T-score	−0.7 ± 0.8	−1.6 ± 0.9	0.043*

*Abbreviations*: *DXA* Dual-energy X-ray absorptiometry, *BMD* Bone mineral density, *LSG* Laparoscopic sleeve gastrectomy, *SD* Standard deviation

Statistically significant at *p* < 0.05 *

When interpreted in relation to least significant change thresholds, the mean postoperative declines in femoral neck and forearm radius bone mineral density in the gastric bypass group exceeded the calculated LSC, supporting that these changes represent true biological loss rather than measurement variability. In contrast, mean changes at the lumbar spine did not consistently exceed LSC thresholds.

Compared to preoperative assessments, a significant reduction in postoperative weight, BMI, and serum calcium level was observed, accompanied by a significant increase in postoperative PTH level (*p* < 0.001), with no significant difference between the two groups. The postoperative serum vitamin D level increased significantly in the sleeve gastrectomy group (*p* = 0.029), with a comparable change observed in the gastric bypass group (Table 4).

**Table 4:** Pre and postoperative anthropometric and laboratory data (*n* = 32)Within-group *P* values represent preoperative versus postoperative comparisons. Between-group *P* values represent comparisons between laparoscopic sleeve gastrectomy and gastric bypass groups

Mean ± SD	LSG (n = 18)	Gastric bypass (n = 14)	*P* value
Preoperative weight (Kg)	134.1±23.8	155.8±36.25	0.05
Postoperative weight (Kg)	81.8±11.1	80.4±10.7	0.70
*P* value	<0.001*	<0.001*	
Preoperative BMI (kg/m²)	50.87±6.89	55.8±10.13	0.10
Postoperative BMI (kg/m²)	31.03±3.47	28.95±3.48	0.10
*P* value	<0.001*	<0.001*	
Preoperative calcium (mg/dL)	9.28±0.3	9.1±0.2	0.06
Postoperative calcium (mg/dL)	8.61±0.4	8.69±0.4	0.57
*P* value	< 0.001*	0.002*	
Preoperative PTH (pg/mL)	47.66±18.6	47±9.7	0.91
Postoperative PTH (pg/mL)	74.93±23.7	73±12.9	0.78
*P* value	< 0.001*	< 0.001*	
Preoperative vitamin D (ng/mL)	26.11±3.9	26±3.4	0.93
Postoperative vitamin D (ng/mL)	29.9±2.2	26.01±6.8	0.029*
*P* value	0.001*	0.99	

*LSG* Laparoscopic Sleeve Gastrectomy, *BMI* Body mass index, *PTH* Parathyroid hormone, *VIT* Vitamin, *n* number, *SD* Standard deviation

^*^Significant at *p* < 0.05

Table 5 shows that among noncompliant patients (*n* = 18), 33.3% had significant bone loss in the spine (*p* = 0.01), 27.8% had significant bone loss in the femoral neck (*p* = 0.03), and 55.5% had significant bone loss in the forearm radius compared to the compliant group (*n* = 14, *p* < 0.001). Serum calcium levels and vitamin D levels were significantly higher in patients who adhered to their medication regimen than in noncompliant patients (9.06 ± 0.3 vs. 8.34 ± 0.16, *p* < 0.001 and 31.7 ± 4.19 vs. 26.74 ± 4.2, *p* = 0.006, respectively). Compliant patients had significantly lower serum PTH levels than non-compliant patients (69.17 ± 12.8 vs. 77.98 ± 10.9, *p* = 0.048).

**Table 5:** Relation between postoperative DXA scan, laboratory tests, and medication compliance (*n* = 32)

		Medication compliance		
Postoperative DXA scan		Yes (n = 14)	No (n = 18)	P value
Spine, n (%)	**Bone loss **	0 (0.0%)	6 (33.3%)	0.01*
	**Normal**	14 (100%)	12 (66.7%)	
Femoral neck, n (%)	**Bone loss **	0 (0%)	5 (27.8%)	0.03*
	**Normal**	14 (100%)	13 (72.2%)	
Forearm (radius), n (%)	**Bone loss **	0 (0.0%)	10 (55.5%)	<0.001*
	**Normal**	14 (100%)	8 (44.5%)	

Postoperative labs		Yes (n = 11)	No (n = 17)	P value
Serum Calcium	**Mean ± SD**	9.06 ± 0.3	8.34 ± 0.16	< 0.001*
PTH	**Mean ± SD**	69.17 ± 12.8	77.98 ± 10.9	0.048*
Vitamin D	**Mean ± SD**	31.7 ± 4.19	26.74 ± 4.2	0.008*

*PTH* Parathyroid hormone, *n* number, *SD* Standard deviation

*Significant at *p*<0.05

Exploratory mixed-design ANOVA demonstrated a significant main effect of time for femoral neck BMD (*p* < 0.05), indicating an overall postoperative decline. The Group × Time interaction showed a trend toward significance but did not reach statistical significance (*p* = 0.08), suggesting a greater decline in the gastric bypass group. These findings are consistent with the pairwise analyses but should be interpreted cautiously due to limited power (Table 6).

**Table 6:** Exploratory 2 × 2 Mixed-Design ANOVA for BMD changes

Skeletal site	Effect	F	*P* value	Partial η²
Lumbar spine	**Main effect of time**	**4.72**	0.038*	**0.13**
	**Main effect of group**	**0.41**	**0.53**	**0.01**
	**Group × time interaction**	**2.11**	**0.156**	**0.07**
Femoral neck	**Main effect of time**	**9.86**	0.004*	**0.25**
	**Main effect of group**	**1.98**	**0.17**	**0.06**
	**Group × time interaction**	**5.62**	0.024*	**0.17**
Forearm (radius)	**Main effect of time**	**8.44**	0.007*	**0.22**
	**Main effect of group**	**2.34**	**0.14**	**0.07**
	**Group × time interaction**	**4.91**	0.033*	**0.15**

Group = LSG vs Gastric Bypass, Time = preoperative vs 12-month postoperative

*BMD* Bone mineral density, η² partial eta squared

*Statistically significant at *p* < 0.05

Metabolic and nutritional assessment included serum calcium, parathyroid hormone (PTH), and 25-hydroxyvitamin D levels measured preoperatively and at 12 months postoperatively. Other micronutrient parameters, including phosphate, magnesium, albumin, vitamin B12, folate, iron indices, and glycemic markers, were not routinely assessed as part of the study protocol.

Early postoperative safety outcomes were assessed at 30 and 90 days. No major surgical complications were observed. Specifically, there were no cases of anastomotic leak, staple-line leak, postoperative bleeding requiring intervention, venous thromboembolism, stricture, or marginal ulcer. No complications of Clavien–Dindo grade III or higher were recorded, and no readmissions or reoperations occurred within the first 90 postoperative days.

## Discussion

Although metabolic and bariatric surgery is a safe and effective intervention for patients with severe obesity, there is a paucity of data concerning its effect on bone density. This study aimed to assess the effect of metabolic and bariatric surgeries on bone mineral density using DXA scans in patients undergoing sleeve gastrectomy or bypass surgery.

An important consideration in interpreting our findings is the heterogeneity of the gastric bypass group, which included both primary and revisional procedures as well as different bypass techniques (LRYGB and OAGB). Revisional bariatric surgery patients may have altered baseline nutritional status and anatomy that could influence postoperative bone metabolism. Nevertheless, the inclusion of revisional cases reflects real-world bariatric practice, where conversion to bypass is commonly performed for weight regain or complications. While subgroup analyses were limited by sample size, the consistent pattern of greater bone loss observed in the bypass group supports the overall conclusion that bypass-type procedures may exert a stronger negative effect on bone density than sleeve gastrectomy.

Our findings show that patients who underwent gastric bypass had a significantly higher risk of bone loss, particularly in the femur neck and radius, while patients who underwent sleeve gastrectomy had a milder impact. In both sleeve gastrectomy and gastric bypass groups, there was a significant decrease in calcium levels and an increase in PTH levels, with no significant difference between the groups in calcium and PTH response. Noncompliant patients had a significantly high incidence of bone loss in the spine, femoral neck, and radius, with a significant decline in serum calcium and vitamin D and a high PTH level.

Similarly, Bredella et al. [18] ascertained that the RYGB group exhibited a greater decrease in total hip and femoral neck BMD compared to the SG group. There was no statistically significant difference between the groups in BMD in the lumbar spine. Shanbhogue et al. [19] observed a significant decrease in mean total hip and spine BMD after 12 months and 24 months. Spine BMD demonstrated no change from 12 to 24, although the mean total hip BMD exhibited a sustained decline. Also, Geoffroy et al. [20] noted that the majority of patients exhibited a clinically significant decrease in BMD at the femoral neck and hip. Moreover, osteopenia was identified at the femoral neck in most of the patients. Lindeman et al. [21] documented a loss of bone mass in weight-bearing bones (e.g., femur and hip). The authors indicated that the average change in BMD was − 9.7% lower in the total hip region, and 12% lower at the femoral neck. At 12 months following sleeve gastrectomy, Azzam et al. [22] found that bone loss was more noticeable at the hip and femoral neck sites than at the lumbar spine. Additionally, BMD declined sharply.

In contrast, Salman et al. [11] observed no significant variations in BMD measurements at the hip, lumbar spine, femoral neck, and the entire body following RYGB and SG operations.

Significant drops in BMD have been associated with weight loss, particularly in older men and postmenopausal women [23, 24]. Because of this, body weight can drop by as much as 30% after bariatric surgery. The reduced mechanical strain resulting from this weight loss can lead to decreased bone formation, increased bone loss, and decreased bone mineral density [25]. However, Ben-Porat et al. [26] showed that bone loss primarily occurs during the period of rapid weight loss and persists long after achieving a stable weight. This suggests that weight loss may not be the only factor contributing to the negative effects of bariatric surgery on bone. Following weight reduction, the skeletal system changes due to various variables and interactions, some of which have unknown effects [27].

Postoperative metabolic changes have a more substantial impact on bone loss compared to conventional weight loss. However, these alterations are accompanied by a decline in food intake, alterations in micronutrient absorption, and other physiological modifications [26]. Previous studies have identified hormonal changes (including gonadal and gastrointestinal hormones), mechanical unloading, and reduced initial absorption of calcium and vitamin D as the primary causes of postoperative bone loss. Additional contributing factors include increased bone marrow fat and decreased lean mass [28]. As indicated by the extant literature, mechanical tension on the bones and joints during motion and exercise has been demonstrated to preserve BMD. This phenomenon can be attributed to the observation that mechanical loading exerts a significant influence on the size, mass, and biomechanical characteristics of human bones [29, 30].

Li et al. [30] posit that osteocytes utilize the sclerostin pathway to locally remodel bone in response to changes in mechanical loading. Weight loss has been demonstrated to reduce skeletal mechanical loading, which, in the absence of exercise or physical activity, can lead to bone deterioration [26]. Consequently, variations in bone density loss between the hip and lumbar spine may be attributable to the differing off-loading of these regions induced by weight loss, as well as by the enhanced degenerative alterations that could influence the assessment of lumbar spinal bone mineral density.

Likewise, Carrasco et al. [31] demonstrated a substantial decrease in calcium levels at 12 and 24 months after SG surgery and RYGB procedure, with no discernible difference in the types of surgeries. Furthermore, Wei J et al. [32] reported that the prevalence of secondary hyperparathyroidism escalated to 35.4% one year and 63.3% five years following surgery.

In contrast, Salman et al. [11] found that the RYGB group had significantly higher PTH concentrations at > 2 years of follow-up compared to the SG group.

Despite the apparent methodological distinctions, both groups have the potential to induce calcium malabsorption. The duodenum and proximal jejunum—the two primary sites for calcium absorption—are circumvented during gastric bypass, resulting in compromised calcium absorption. Given the pH-dependence of calcium absorption, LSG has been shown to reduce gastric acid output, thereby impacting calcium solubility and absorption [33].

In the current study, postoperative calcium levels did not significantly differ between the two types of surgeries, which explains the observed similarity in PTH outcomes between the two groups. Additionally, the follow-up period of our study is relatively brief. However, PTH levels continue to increase after the first year following surgery, particularly in patients who underwent gastric bypass surgery due to chronic malabsorption.

Furthermore, the sleeve gastrectomy group patients exhibited a significant increase in vitamin D levels compared to the gastric bypass group. This finding suggests that sleeve gastrectomy may have a greater positive impact on vitamin D status after surgery. Similarly, Geoffroy et al. [20] determined that more than half of bariatric surgery patients had a vitamin D level more than 30 ng/mL at 6 months and 12 months, with 0.9% of patients experiencing a fracture following surgery. Moreover, Markopoulos et al. [34] revealed no significant discrepancy between the SG and RYGB groups concerning the alterations in Ca and vitamin D. However, Bilezikian et al., [35] observed no significant variations in serum calcium or vitamin D levels between the two groups post-surgery.

Our findings revealed that patients who were adherent to the treatment regimen demonstrated significantly less bone loss and high calcium and vitamin D levels and low PTH levels. This finding aligns with Muschitz et al. [36], who noted that the loss of BMD post-bariatric surgery was mitigated by the administration of vitamin D, calcium, and protein supplementation, adjusted for BMI, in conjunction with exercise regimens.

Leong, et al. [37] noted a significant correlation between the extent of serum calcium decline and a reduction in bone mineral density at the femoral neck. On the other hand, Spetz et al. [38] observed that, except for a 10% prevalence of anemia, vitamin D, serum calcium levels, vitamin B12, and iron were uncommon two years post-surgery in both compliant and noncompliant patients.

Baseline differences between groups should be considered when interpreting our findings. Notably, type 2 diabetes mellitus was present only in the gastric bypass group. Therefore, while diabetes may influence skeletal fragility through mechanisms not fully captured by BMD, its impact on the present DXA-based outcomes is likely limited. Nonetheless, residual confounding cannot be completely excluded.

The recommendation for early bone health monitoring is based on observed changes in bone mineral density and mineral metabolism rather than fracture outcomes or pharmacologic intervention.

Incorporation of DXA precision error and least significant change supports the robustness of the observed bone loss at appendicular sites, particularly following bypass-type procedures.

Although exploratory mixed ANOVA was performed, the study was not powered to reliably detect interaction effects; therefore, conclusions regarding differential trajectories should be considered hypothesis-generating.

The relatively small sample size limits the statistical power to detect modest between-group differences in bone mineral density, particularly across multiple skeletal sites. As a result, the risk of Type II error cannot be excluded. While significant differences were observed at the femoral neck and forearm radius, the absence of statistically significant differences at other sites does not necessarily indicate equivalence between procedures.

### Limitations

This study was conducted at a single center with a relatively small sample size, which may limit generalizability. In addition, follow-up was limited to one year, precluding assessment of longer-term skeletal outcomes.

The gastric bypass cohort was heterogeneous, including both primary and revisional procedures and different bypass techniques, which may have introduced confounding and limited the ability to isolate procedure-specific effects. Baseline imbalances, including the presence of type 2 diabetes mellitus exclusively in the gastric bypass group, represent additional potential confounders. The small sample size also limited the feasibility of robust multivariable adjustment.

Medication compliance was assessed using patient self-report rather than objective measures, which may have introduced recall or reporting bias. Furthermore, dietary intake, physical activity, sun exposure, and comprehensive postoperative assessment of all micronutrient and metabolic parameters were not evaluated, which may limit interpretation of broader nutritional and metabolic effects.

Weight-related outcomes were reported descriptively, and the study did not evaluate prescribed or targeted weight loss. Larger multicenter studies with stratified analyses and longer follow-up are needed to further understanding of bone health changes after bariatric surgery.

## Conclusions

A greater risk of postoperative bone loss has been associated with gastric bypass compared to sleeve gastrectomy. Adherence to medication regimens has been demonstrated to play a pivotal role in mitigating this risk by ensuring the maintenance of optimal calcium and vitamin D levels, as well as preventing PTH elevation. These findings underscore the significance of meticulous monitoring, supplementation, and patient education on adherence to ensure optimal bone health outcomes following metabolic and bariatric surgery.

## Data Availability

The data supporting the findings of this study are contained within the article.

## References

[1] Wang Y, Shirore RM, Ramakrishnan C, Tan NC, Jafar TH. Adiposity measures and pre-diabetes or diabetes in adults with hypertension in Singapore polyclinics. J Clin Hypertens (Greenwich). 2019;21:953–62. 31222909 10.1111/jch.13587 PMC8030616

[2] Coroyannakis C, Khalil A. Management of hypertension in the obese pregnant patient. Curr Hypertens Rep. 2019;21:24. 30915600 10.1007/s11906-019-0927-x PMC6435623

[3] Thaher O, Wollenhaupt F, Croner RS, Hukauf M, Stroh C. Evaluation of the effect of sleeve gastrectomy versus Roux-en-Y gastric bypass in patients with morbid obesity: a multicenter comparative study. Langenbecks Arch Surg. 2024;409:156. 38730065 10.1007/s00423-024-03341-9 PMC11087333

[4] Soong TC, Lee MH, Lee WJ, Almalki OM, Chen JC, Wu CC, et al. Long-term efficacy of bariatric surgery for the treatment of super-obesity: comparison of SG, RYGB, and OAGB. Obes Surg. 2021;31:3391–9. 33993423 10.1007/s11695-021-05464-0

[5] Rousseau C, Jean S, Gamache P, Lebel S, Mac-Way F, Biertho L, et al. Change in fracture risk and fracture pattern after bariatric surgery: a nested case-control study. BMJ. 2016;354:i3794. 27814663 10.1136/bmj.i3794 PMC4964103

[6] Mele C, Caputo M, Ferrero A, Daffara T, Cavigiolo B, Spadaccini D, et al. Bone response to weight loss following bariatric surgery. Front Endocrinol (Lausanne). 2022;13:921353. 35873004 10.3389/fendo.2022.921353 PMC9301317

[7] Ou X, Chen M, Xu L, Lin W, Huang H, Chen G, et al. Changes in bone mineral density after bariatric surgery in patients of different ages or postoperative periods: a systematic review and meta-analysis. Eur J Med Res. 2022;27:144. 35934692 10.1186/s40001-022-00774-0 PMC9358806

[8] McPhee C, Aninye IO, Horan L. Recommendations for improving women’s bone health throughout the lifespan. J Womens Health (Larchmt). 2022;31:1671–6. 36346282 10.1089/jwh.2022.0361 PMC9805882

[9] Osterhoff G, Morgan EF, Shefelbine SJ, Karim L, McNamara LM, Augat P. Bone mechanical properties and changes with osteoporosis. Injury. 2016;47(Suppl 2):S11–20. 27338221 10.1016/S0020-1383(16)47003-8 PMC4955555

[10] You H, Shang J, Huang Z, He W, Zeng C, Xu H, et al. DXA bone density measurements and trabecular bone scores in Chinese adults with obesity before and after bariatric surgery. Sci Rep. 2024;14:29355. 39592749 10.1038/s41598-024-80107-9 PMC11599751

[11] Salman MA, Aradaib M, Salman A, Elewa A, Tourky M, Shaaban HE. Effects of gastric bypass and sleeve gastrectomy on bone mineral density and bone turnover markers: a systematic review and meta-analysis. World J Surg. 2022;46:865–75. 35006326 10.1007/s00268-021-06429-1

[12] Aaseth JO, Alexander J. Postoperative Osteoporosis in Subjects with Morbid Obesity Undergoing Bariatric Surgery with Gastric Bypass or Sleeve Gastrectomy. Nutrients. 2023;15(6):1302. 10.3390/nu15061302. PMID: 36986032; PMCID: PMC10057453. 10.3390/nu15061302 PMC10057453 36986032

[13] Ebadinejad A, Ahmadi AR, Ghazy F, Barzin M, Khalaj A, Valizadeh M, et al. Changes in bone turnover markers after Roux-en-Y gastric bypass versus sleeve gastrectomy: a systematic review and meta-analysis. Obes Surg. 2023;33:1259–69. 36790646 10.1007/s11695-023-06503-8

[14] Krugh M, Langaker MD. Dual-energy X-ray absorptiometry. In: StatPearls. Treasure Island (FL): StatPearls Publishing; 2025. 30085584

[15] Lewiecki EM. Premenopausal bone health assessment. Curr Rheumatol Rep. 2005;7:46–52. 15760580 10.1007/s11926-005-0008-9

[16] Abraham A, Cohen A, Shane E. Premenopausal bone health: osteoporosis in premenopausal women. Clin Obstet Gynecol. 2013;56:722–9. 24022503 10.1097/GRF.0b013e3182a8ae55 PMC4139057

[17] Misra M, Animashaun A, Bose A, Singhal V, Stanford FC, Carmine B, et al. Impact of sleeve gastrectomy on hip structural analysis in adolescents and young adults with obesity. Surg Obes Relat Dis. 2020;16:2022–30. 32861645 10.1016/j.soard.2020.07.020 PMC7704626

[18] Bredella MA, Greenblatt LB, Eajazi A, Torriani M, Yu EW. Effects of Roux-en-Y gastric bypass and sleeve gastrectomy on bone mineral density and marrow adipose tissue. Bone. 2017;95:85–90. 27871812 10.1016/j.bone.2016.11.014 PMC5222731

[19] Shanbhogue VV, Støving RK, Frederiksen KH, Hanson S, Brixen K, Gram J, et al. Bone structural changes after gastric bypass surgery evaluated by HR-pQCT: a two-year longitudinal study. Eur J Endocrinol. 2017;176:685–93. 28289103 10.1530/EJE-17-0014 PMC5425940

[20] Geoffroy M, Charlot-Lambrecht I, Chrusciel J, Gaubil-Kaladjian I, Diaz-Cives A, Eschard JP, et al. Impact of bariatric surgery on bone mineral density: an observational study of 110 patients. Obes Surg. 2019;29:1765–72. 30734230 10.1007/s11695-019-03719-5

[21] Lindeman KG, Rushin CC, Cheney MC, Bouxsein ML, Hutter MM, Yu EW. Bone density and trabecular morphology at least 10 years after gastric bypass and gastric banding. J Bone Miner Res. 2020;35:2132–42. 32663365 10.1002/jbmr.4112

[22] Azzam AI, Ghanem S, Elzayat A. The short-term impact of sleeve gastrectomy on bone health. Mediterr J Rheumatol. 2025;36:86–91. 40557180 10.31138/mjr.030324.tst PMC12183443

[23] Ensrud KE, Fullman RL, Barrett-Connor E, Cauley JA, Stefanick ML, Fink HA, et al. Voluntary weight reduction in older men increases hip bone loss. J Clin Endocrinol Metab. 2005;90:1998–2004. 15671096 10.1210/jc.2004-1805

[24] Von Thun NL, Sukumar D, Heymsfield SB, Shapses SA. Does bone loss begin after weight loss ends? Menopause. 2014;21:501–8. 24149920 10.1097/GME.0b013e3182a76fd5 PMC5032655

[25] Corbeels K, Verlinden L, Lannoo M, Simoens C, Matthys C, Verstuyf A, et al. Thin bones: vitamin D and calcium handling after bariatric surgery. Bone Rep. 2018;8:57–63. 29955623 10.1016/j.bonr.2018.02.002 PMC6019966

[26] Ben-Porat T, Elazary R, Sherf-Dagan S, Goldenshluger A, Brodie R, Mintz Y, et al. Bone health following bariatric surgery: implications for management strategies. Adv Nutr. 2018;9:114–27. 29659692 10.1093/advances/nmx024 PMC5916426

[27] Williams SE, Cooper K, Richmond B, Schauer P. Perioperative management of bariatric surgery patients: focus on metabolic bone disease. Cleve Clin J Med. 2008;75:333–48. 18556875 10.3949/ccjm.75.5.333

[28] Saad R, Habli D, El Sabbagh R, Chakhtoura M. Bone health following bariatric surgery: an update. J Clin Densitom. 2020;23:165–81. 31519474 10.1016/j.jocd.2019.08.002

[29] Villareal DT, Fontana L, Weiss EP, Racette SB, Steger-May K, Schechtman KB, et al. Bone mineral density response to caloric restriction- or exercise-induced weight loss. Arch Intern Med. 2006;166:2502–10. 17159017 10.1001/archinte.166.22.2502

[30] Li W, Lin D, Chen J, Zhang Z, Liao Z, Swain M, et al. Role of mechanical stimuli in oral implantation. J Biosci Med. 2014;2:63–8.

[31] Carrasco F, Basfi-Fer K, Rojas P, Csendes A, Papapietro K, Codoceo J, et al. Calcium absorption after sleeve gastrectomy or Roux-en-Y gastric bypass in premenopausal women. Am J Clin Nutr. 2018;108:24–32. 29878034 10.1093/ajcn/nqy071

[32] Wei JH, Lee WJ, Chong K, Lee YC, Chen SC, Huang PH, et al. High incidence of secondary hyperparathyroidism in bariatric patients. Obes Surg. 2018;28:798–804. 28921422 10.1007/s11695-017-2932-y

[33] Schafer AL, Weaver CM, Black DM, Wheeler AL, Chang H, Szefc GV, et al. <article-title update="added">Intestinal calcium absorption decreases dramatically after gastric bypass surgery despite optimization of vitamin D status. J Bone Miner Res. 2015;30:1377–85. 25640580 10.1002/jbmr.2467 PMC4593653

[34] Markopoulos G, Skroubis G, Kalfarentzos F, Kehagias I. Comparison of one-anastomosis gastric bypass versus Roux-en-Y gastric bypass. Prz Gastroenterol. 2022;17:152–61. 35664023 10.5114/pg.2021.108453 PMC9165332

[35] Bilezikian J, Raisz L, Martin T. Principles of Bone Biology. 2nd ed. Academic Press; 2008.

[36] Muschitz C, Kocijan R, Haschka J, Zendeli A, Pirker T, Geiger C, et al. Impact of vitamin D, calcium, protein supplementation, and exercise on bone metabolism after bariatric surgery. J Bone Min Res. 2016;31:672–82. 10.1002/jbmr.2707 26350034

[37] Ieong K, Ardila-Gatas J, Yang J, Zhang X, Tsui ST, Spaniolas K, et al. Bone mineral density changes after bariatric surgery. Surg Endosc. 2021;35:4763–70. 32909203 10.1007/s00464-020-07953-2

[38] Spetz K, Svedjeholm S, Roos S, Grehn S, Olbers T, Andersson E. Adherence to vitamin and mineral supplementation after bariatric surgery. Obes Res Clin Pract. 2022;16:407–12. 36151032 10.1016/j.orcp.2022.09.001

